# The Role of Molecular Testing for the Indeterminate Thyroid FNA

**DOI:** 10.3390/genes10100736

**Published:** 2019-09-23

**Authors:** Esther Diana Rossi, Liron Pantanowitz, William C. Faquin

**Affiliations:** 1 Division of Anatomic Pathology and Histology, Catholic University of Sacred Heart, 00168 Rome, Italy; esther.rossi@policlinicogemelli.it; 2 Department of Pathology, University of Pittsburgh Medical Center, Pittsburgh, PA 15232, USA; pantanowitzl@upmc.edu; 3 Department of Pathology, Massachusetts General Hospital and Harvard Medical School, Boston, MA 02114, USA

**Keywords:** fine needle aspiration, cytology, thyroid cancers, molecular testing, personalized medicine

## Abstract

Thyroid nodules are common in the adult population where a majority are benign and only 4.0% to 6.5% are malignant. Fine needle aspiration (FNA) is a key method used in the early stages to evaluate and triage patients with thyroid nodules. While a definitive cytological diagnosis is provided in more than 70–75% of all thyroid FNA cases, the group of indeterminate lesions offers a challenge in terms of interpretation and clinical management. Molecular testing platforms have been developed, are recognized as an option by the 2015 American Thyroid Association Guidelines, and are frequently used in conjunction with FNA as an integral part of the cytologic evaluation. In this review, the utility of molecular testing options for nodules assigned to the group of indeterminate thyroid FNAs is described.

## 1. Introduction

Fine-needle aspiration (FNA) is an important diagnostic tool for the initial evaluation of thyroid nodules because of its overall simplicity, safety, accuracy, and cost-effectiveness [1,2,3,4]. Approximately 70% of thyroid nodules are benign with only 5–10% reported as “malignant” [1,2,3,4,5,6]. The remaining 15–30% of thyroid FNAs are classified using the Bethesda System for Reporting Thyroid Cytopathology (TBSRTC) in the so-called “grey zone of indeterminate proliferations” for which a cytomorphological discrimination is not possible [7,8,9,10,11,12,13,14,15,16,17,18]. Thyroid nodules classified as indeterminate are the leading challenge for both cytopathologists and treating clinicians since the indeterminate diagnostic categories can lead to unnecessary surgical interventions and higher health care costs in almost 20–25% of indeterminate lesions.

The application of molecular analysis for thyroid FNA specimens was accepted in 2015 by the American Thyroid Association as well as other clinical endocrinology groups as a follow-up option for thyroid FNAs classified into one of the indeterminate categories [7,18]. The objective of molecular testing would be to provide more definitive guidance for the treating clinician to assist with decision making with regard to the management approach. Over the past decade, numerous studies have demonstrated the high sensitivity and overall diagnostic accuracy of molecular testing when applied to cytologic specimens [19,20,21,22,23,24,25,26,27,28,29,30,31,32,33,34,35,36,37,38,39]. In fact, the application of ancillary molecular analysis is not unique to thyroid cytopathology, but it has become an integral part of the management of several different tumors such as lung and others. As molecular approaches continue to be improved, the molecular analysis of various tumors can be translated into clinical practice as an adjuvant tool for diagnosis, clinical management, specialized therapy, and prognosis [40].

While aspects of molecular testing were not sufficiently refined and vetted to be included in the first edition of TBSRTC, the role of molecular testing to assist in guiding management decisions including with the recently described non-invasive follicular thyroid neoplasm with papillary-like nuclear features (NIFTP), was included in the recent second edition of TBSRTC [41,42,43]. This review provides an overview of the key role that molecular testing is increasingly playing in the evaluation of thyroid FNA specimens, in particular those classified within the indeterminate categories of TBSRTC.

## 2. Molecular Testing and the Indeterminate Categories of the Bethesda System

In the USA, thyroid cancer (TC) accounts for 6% of cancers in women and less than 3% of cancers in men. Papillary TC (PTC) and Follicular TC (FTC) arise from follicular cells, and they constitute approximately 90% of all TC [1,2,3,4]. Data from the literature show that although TC generally has a very good prognosis, 10–15% of TCs are associated with recurrences and metastases to regional lymph nodes or distant sites including about 5% of patients with TCs that are not responsive to radioactive-iodine (RAI) and who may eventually die from their disease. For this reason, early diagnosis of TC and proper triaging of patients with thyroid nodules is needed. FNA plays a key role in differentiating benign from malignant thyroid nodules. However, morphology alone is not able to solve all of the diagnostic questions and the application of ancillary techniques, in particular molecular testing, is being increasingly used to improve the overall performance of FNA [42,43,44,45,46,47].

In 2014, the Thyroid Cancer Genome Atlas published findings that clarified our knowledge of the molecular pathology for PTC. The study examined a large cohort of PTCs and concluded that they can be separated into 2 distinct broad categories, those PTCs with a *BRAF^V600E^-like* profile and those that are *RAS*-like. In addition, most common mutations are shown to activate the mitogenic-activated protein kinase (MAPK) pathway in PTC [40]. In a meta-analysis by Trimboli et al., the authors found that a very low rate of lesions with indeterminate cytology are BRAF mutated. Thus, the role of this biomarker to detect or exclude cancers in patients with FNA reports of indeterminate lesions is limited [4]

As alluded to previously, in 2015, the American Thyroid Association (ATA) published revised management guidelines for patients with thyroid nodules and differentiated TCs (DTC) and recommended the use of molecular diagnostic as an option in FNA cases with an indeterminate cytology [7,19]. Specifically, the ATA Guidelines suggest that the performance of molecular panels (including markers such as *BRAF, RAS, RET/PTC and PAX8-PPARγ*) can significantly improve the accuracy of preoperative FNA in patients with indeterminate thyroid FNA samples. Furthermore, molecular testing could be used to stratify the risk of malignancy (ROM) of a particular indeterminate thyroid FNA, and when applied to those FNAs with a high negative predictive value (NPV), patients might avoid unnecessary surgical intervention, either lobectomy or total thyroidectomy. The 2015 ATA Guidelines did not endorse any specific molecular test, but, the recent 2nd edition of TBSRTC has suggested the potential role of different molecular tests in selected diagnostic categories and diagnostic scenarios. However, Ferris et al., in their ATA statement on surgical application of molecular profiling for thyroid nodules, did offer a suggestion for differential use of molecular testing in the diagnostic subcategories of TBSRTC indeterminate lesions. [19]. Familiarity with molecular testing options available for thyroid cytopathology as well as their indications, significance, and limitations is an important aspect of thyroid cytopathology, and may have implications for NIFTP [47,48,49,50,51,52,53,54,55,56,57,58,59,60].

Numerous studies have demonstrated that the detection of specific somatic mutations, gene rearrangements, and/or microRNA (miRNA) expression profiles can have high specificity and high positive predictive values for malignant thyroid disease [61,62,63,64,65,66,67,68,69,70,71,72,73,74,75,76,77,78,79]. The use of molecular testing panels rather than single gene mutation assays has provided the most successful results [61,62,63,64,65,66,67]. For example, Nikiforov et al., demonstrated the advantage of using a broad next-generation sequencing (NGS) panel that provides a highly accurate, informative and more comprehensive analysis of somatic mutations and chromosomal rearrangements in the diagnosis of nodules with AUS/FLUS and FN/SFN cytology, which ultimately facilitates the optimal management of these patients [68,69,70,71,72,73,74,75,76,77,78,79,80,81,82].

Despite the fact that currently there is no single, unequivocal molecular approach for the cytological evaluation of thyroid nodules, several molecular thyroid tests are commercially available in the USA including: a: ThyroSeq.v3 (University of Pittsburgh Medical Center [UPMC]/Cytopath Biopsy Lab [CBLPath], Pittsburgh, PA, USA); b: Afirma Gene Sequencing Classifier and Xpression Atlas (GSC & XA, Veracyte, South San Francisco, CA, USA); and c: ThyGenX and ThyraMIR (both from Interpace Diagnostics, Parsippany, NJ) [82,83]. These tests offer high NPV’s as well as reasonable positive predictive values (PPV) for the evaluation of indeterminate thyroid FNA cases [84,85,86,87,88,89,90,91,92,93,94,95,96,97,98,99,100,101,102]. The acknowledgement of the role of molecular testing formalizes its use in defining risk stratification of patients with thyroid nodules. 

### 2.1. AUS/FLUS

The AUS/FLUS diagnostic category of TBSRTC was endorsed in the 2017 2nd edition of TBSRTC with the addition of only minor changes. The category continues to pose diagnostic challenges for cytopathologists and management challenges for treating clinicians. The AUS/FLUS category is often the subject of investigation of ancillary studies to improve the triage of patients receiving this diagnosis.

**Figure 1:**
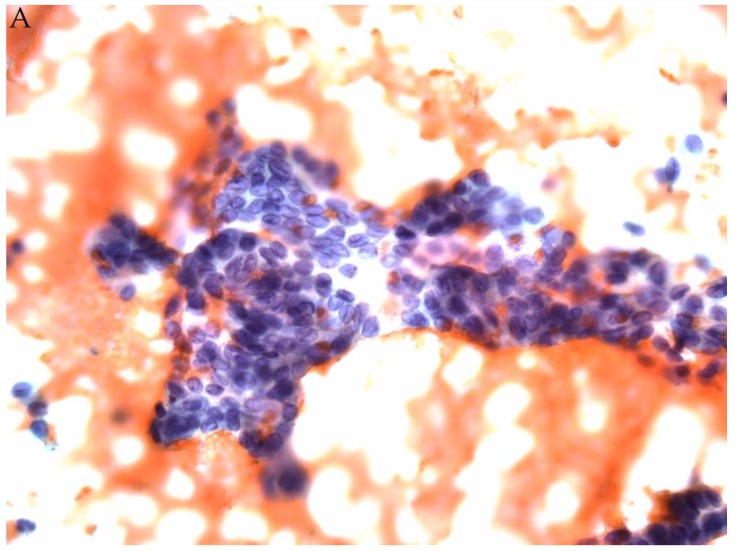

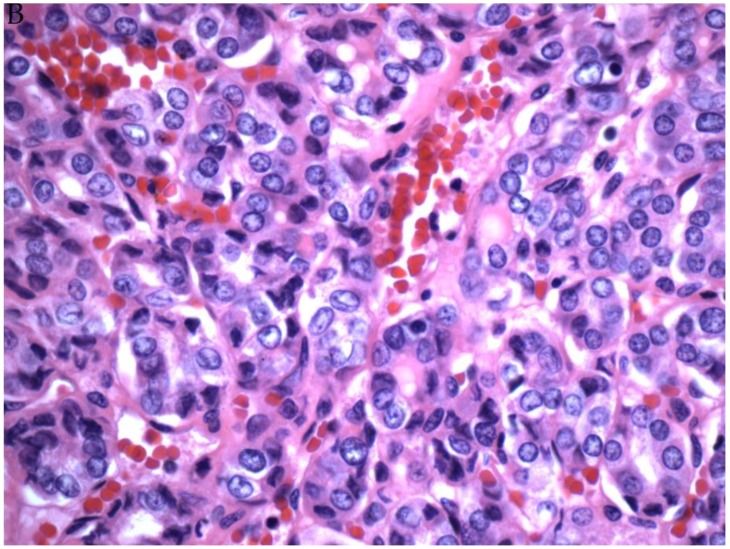
(**A**) FNA of a thyroid nodule classified as AUS/FLUS (Papanicolaou stain). Afirma testing was Suspicious. (**B**) Corresponding histology showed an encapsulated follicular variant of papillary thyroid carcinoma (H&E stain).

In agreement with the 2015 ATA guidelines, an initial AUS/FLUS interpretation is typically followed by either repeat FNA or molecular testing [7]. Over 50% of nodules initially diagnosed as AUS/FLUS would be reclassified as Benign by repeat FNA; however, approximately 10–30% of cases would be classified as AUS/FLUS by repeat FNA. In those cases and other scenarios with an AUS/FLUS classification, the application of mutational testing could provide additional diagnostic information that could impact clinical management decisions including clinical follow-up versus surgical resection (Figure 1). 

The second edition of suggests that decisions regarding surgery (typically lobectomy) vs. continued observation should be based on a combination of cytologic, molecular, clinical, and radiologic findings that cannot exclude the evaluation of clinical risk factors and patient preferences [41]. It is also worth noting that the ROM associated with a thyroid nodule classified as AUS/FLUS varies significantly with the subtype of AUS/FLUS, ranging from a mean ROM of 47% for AUS/FLUS with cytologic atypia to only 5% for AUS/FLUS with Hürthle cell features [6,7,8,9,10,11].

Several studies have confirmed that the AUS/FLUS category represents a low risk of malignancy and, when malignant, the follow-up histological diagnosis is most often FVPTC, so that an expanded mutation panel might have higher sensitivity than *BRAF^V600E^* alone but diminished specificity due to the increased prevalence of *RAS* mutations.

In 2007, Nikiforov et al., developed a 7-gene molecular test (ThyroSeq v0), composed of a panel of mutations (*BRAF, N-/H-/K-RAS*) as well as the translocations *RET/PTC* and *PAX8/PPAR* [32]. The authors included a series of 1056 indeterminate thyroid lesions, classified according to TBSRTC, and they found an increased ROM for mutated AUS/FLUS, FN, and SM cases (88%, 87%, and 95% respectively), compared to 6%, 14%, and 28% in mutation-negative thyroid nodules [32]. Among the 1056 indeterminate lesions, the cohort included 653 cytological cases of AUS/FLUS with 247 that had histological follow-up. The detection of any mutations increased the risk of cancer from 14% to 87%, whereas the absence of any mutations was linked with a cancer risk of 6%.

In 2013, Nikiforov et al., began applying next generation sequencing (NGS) technology as a new approach for testing a broad spectrum of point mutations [34]. In 2014, the first mutational panel, ThyroSeq v1 including 15-genes was followed by a new and superior NGS-based assay, ThyroSeq v2, that was applied initially to 143 cases of FN/SFN with the evaluation of an expanded 56-gene panel to include several point mutations and gene fusions with high NPV [35]. In 2015, Nikiforov et al., evaluated the impact of ThyroSeq v2 on the AUS/FLUS category showing that the test performance was dependent on the pre-test probability of malignancy for this category [36]. Their analysis demonstrated a sensitivity of 90.9%, specificity of 92.1%, PPV of 76.9%, and NPV of 97.2% with an overall accuracy of 91.8%. The disease prevalence was in agreement with the Bethesda diagnostic categories.

In 2017, the same group released their most recent version, the ThyroSeq v.3 test, which includes more than 12000 mutation hotspots and more than 120 gene fusion types. In a recent prospective study, including 10 medical centers with 286 cytologically indeterminate lesions, Steward et al., found that in the Bethesda III and IV categories combined, ThyroSeq v.3 demonstrated a 94% sensitivity and 82% specificity [84]. The conclusion is that application of ThyroSeq v.3 could potentially obviate surgery in up to 61% of those patients with an indeterminate thyroid cytology [84].

The Afirma Gene Expression Classifier (GEC) is a commonly used molecular test for indeterminate thyroid proliferations based on the concept of predicting benign thyroid lesions [37,85,86,87,88,89,90,91]. In 2012, the Afirma GEC was validated in a key study published by Alexander et al., including 265 indeterminate thyroid lesions out of 4812 FNA cases from a multicenter trial [37]. The original Afirma test was based on the expression of 167 genes including 142 genes in the main classifier (benign or suspicious) and 25 smaller gene expression panels to filter out rare neoplasms [37]. Alexander and colleagues in 2012 demonstrated a 95% NPV for AUS/FLUS lesions and a 94% NPV for FN with an associated malignancy rate of 24% and 25%, respectively [37]. Of note, the authors concluded that the Afirma GEC identified thyroid nodules as benign with a very low ROM in the AUS/FLUS and SFN/FN categories. In contrast, the test’s NPV was much lower for the suspicious for malignancy (SFM) category indicating that it would not be as useful for nodules classified as SFM.

Since its initial introduction, many published studies have demonstrated the utility of the Afirma GEC for AUS/FLUS lesions. The data confirm that approximately one-half of AUS/FLUS cases have a “Benign” GEC result, and that it is more frequently ascribed to those AUS/FLUS cases with isolated architectural atypia than those AUS/FLUS cases with nuclear atypia or nuclear plus architectural atypia. Specifically, the rate of Benign GEC results was higher for AUS/FLUS thyroid nodules with architectural atypia (65%) than in AUS/FLUS nodules with nuclear atypia (59%) or AUS/FLUS with both nuclear and architectural atypia (38%). In patients who had GEC Suspicious nodules, the ROM is higher in cases with both architectural and nuclear atypia (57%) than in cases with architectural or nuclear atypia alone (19% and 45% respectively) [87]. Based on evidence that a benign result is associated with a ROM decreasing from 24% to 5%, observation over surgery is considered an appropriate choice for patients with a Benign GEC test result [48].

Recently, a next-generation Afirma Genomic Sequencing Classifier (GSC) was introduced as a new advancement. The Afirma GSC combines not only gene expression, but also the presence of DNA variants, fusions, copy number variants, and other information that may be predictive of thyroid cancer. Specifically, the Afirma GSC does not use expression pattern (RNA microarray) but it uses RNA-seq analysis [92]. The purpose of GSC is to maintain the high original test sensitivity while also significantly increasing its specificity. This results in approximately 70% of patients with indeterminate cytology avoiding unnecessary surgery [92,93]. Endo et al., from a single academic tertiary center, found an improved specificity and PPV while maintaining high sensitivity and NPV for GSC compared with GEC. A statistically significant increase in benign call rates was observed in GSC compared with GEC, likely indicating fewer false positive results. After implementation of the Afirma GSC, surgical interventions have been reduced by 68% [94].

### 2.2. Follicular Neoplasm (FN/SFN)

The FN/SFN diagnostic category is another indeterminate category of TBSRTC where the application of molecular testing has been useful. In a study analyzing the use of Thyroseq v0, Nikiforov et al., included 247 FN/SFN cases with 214 having histological follow-up [32]. Among the 38 resected nodules that were associated with a defined molecular alteration, 33 (87%) were found to be histologically malignant, and all *BRAF* and *PAX8/PPAR*γ-positive nodules were malignant [32]. The ThyroSeq mutational analysis from these FNA studies was associated with 57% sensitivity, 97% specificity, 86% diagnostic accuracy, 87% PPV and 86% NPV [32]. 

**Figure 2:**
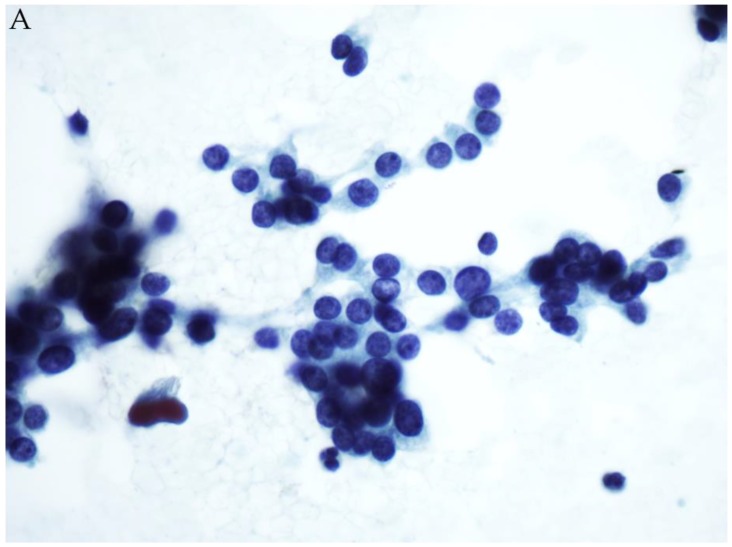

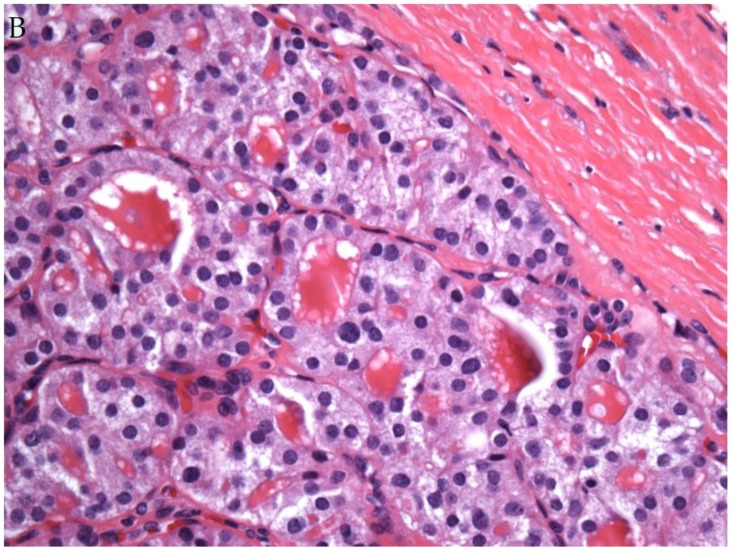
(**A**) FNA of a thyroid nodule classified as FN/SFN (Papanicolaou stain). ThyroSeq v3 testing showed an H-RAS mutation. (**B**) Corresponding histology showed a follicular adenoma (H&E stain).

In 2014, the analysis of 143 FN/SFN with ThyroSeq v2 in a series of 143 retrospectively and prospectively collected FN/SFN nodules showed a performance of 90% sensitivity, 93% specificity, 83% PPV and 96% NPV [35]. The authors confirmed in this series that point mutations in *BRAF^V600E^*, *TERT, TP53, PIK3CA* and any gene fusion were associated with cancer in 100% of thyroid FNA cases [35]. The results of this study indicate that, due to its high PPV and NPV, ThyroSeq v2 and the more recent ThyroSeq v3 are essentially able to perform as both “rule-out” and “rule-in” tests for the FN/SFN category (Figure 2) [35,65,66,67,68,69,70,71,72,73,74,75,76,95,96,97,98,99]. While the application of molecular testing identifying point mutations and rearrangements was proving to be useful for identifying those FN/SFNs with a microfollicular architecture, there were signs that its ability to distinguish Hurthle cell carcinomas from Hurthle cell adenomas was more challenging. However, these issues were addressed by Thyroseq v3, which includes assessment of gene copy number variations, a feature useful in the evaluation of Hurthle cell neoplasms. The Afirma GSC test also addressed the problem of overclassifying Hurthle cell lesions as Suspicious. Several studies reported a lower specificity or higher false positive rate in GEC tests among indeterminate nodules with Hurthle cell predominance. Brauner et al., included a cohort of 122 Hurthle cell-predominant nodules identified as GEC Suspicious with a corresponding benign histopathology [95]. Nonetheless, the development of the Afirma GSC was able to improve the test performance for Hürthle-cell lesions with increased specificity of 59% compared with just 12% with the original Afirma GEC [92,93].

Other authors found that RosettaGX Reveal, which was a microRNA-based diagnostic assay, was also used to further evaluate cytologically indeterminate thyroid nodules and that it might prevent over 75% of unnecessary surgeries for initial indeterminate diagnoses. Lithwick-Yanai et al., using the microRNA-based assay for the AUS/FLUS and FN/SFN categories, showed that the sensitivity and specificity were both 74%, with a NPV of 92% and PPV of 43% [94]. RosettaGXReveal had the advantage of using FNA material obtained by scraping archival cytology slides. Although having several promising advantages, the test is unfortunately no longer commercially available.

Another molecular testing option which has received some attention is ThyGenX (Interpace Diagnostics, Parsippany, NJ), a thyroid 8-gene panel representing a “newer version” of the original gene panel test to detect genetic alterations. This is another NGS-based technology, commercially known as miRInform (Asuragen, Austin, Tex, USA). Based on its specific methodology, the detection of *BRAF^V600E^* or *RET/PTC* is associated with 100% ROM, but the ROM is lower and wider for *RAS mutations* (range, 12–87.5%) and *PAX8/PPARg* rearrangements (range, 50–100%). The ROM for wild type AUS/FLUS is only slightly higher than that of an FNA classified as benign while the ROM for a wild type FN/SFN is identical to the non-tested cases. 

Based on these results, Interpace Diagnostics propose ThyraMIR (from Interpace Diagnostics, Parsippany, NJ, USA) as an additional reflex test, for those cases with a wild type/negative ThyGenX result [96,97]. ThyraMIR is a thyroid microRNA (miRNA) classifier that is able to divide results into “positive” or “negative” categories. Different studies have demonstrated the successful application of miRNA detection on different cytological material, including indeterminate thyroid FNA lesions. Aberrant expression of specific miRNAs (e.g., miR-146, 221, 222) are considered a clue to thyroid well differentiated carcinomas [65,66,67,68,69,70,71,72,73,74,75,76].

High sensitivity and specificity are obtained using the combination testing of ThyGenX and ThyraMIR as demonstrated in two different studies that combined these tests for indeterminate thyroid nodules [96,97]. They demonstrated high sensitivity (94% for AUS/FLUS and 82% for SFN/FN) and specificity (80% for AUS/FLUS and 91% for SFN/FN), with a PPV of 74% and NPV of 94% [96,97]. The application of multi-panel testing not only provides important information about specific mutations being present, but also the prognostic relevance of some of these mutations that would suggest management option such as a total thyroidectomy versus lobectomy [96,97].

### 2.3. The Application of Molecular Testing to NIFTP

The new terminology of “noninvasive follicular thyroid neoplasm with papillary-like nuclear features” (NIFTP) was introduced to replace the encapsulated-noninvasive follicular variant of PTC (FVPTC). NIFTPs are biologically similar to follicular adenomas, lacking features of carcinoma such as lymph node metastasis and/or recurrence. NIFTP is a histological diagnosis defined by strict major and minor histological criteria. FNA cannot be used to specifically diagnose NIFTP because the diagnosis is made based on encapsulation of the tumor. However, NIFTP has had significant impact on thyroid cytology including on the ROM of the different diagnostic categories in TBSRTC. A wide range of studies have demonstrated that the majority of NIFTP are classified cytologically in the indeterminate categories with 31% in the AUS/FLUS, 26.6% in the FN/SFN and 24.3% in the SFM [56,57,58,59,62,63,64].

**Figure 3:**
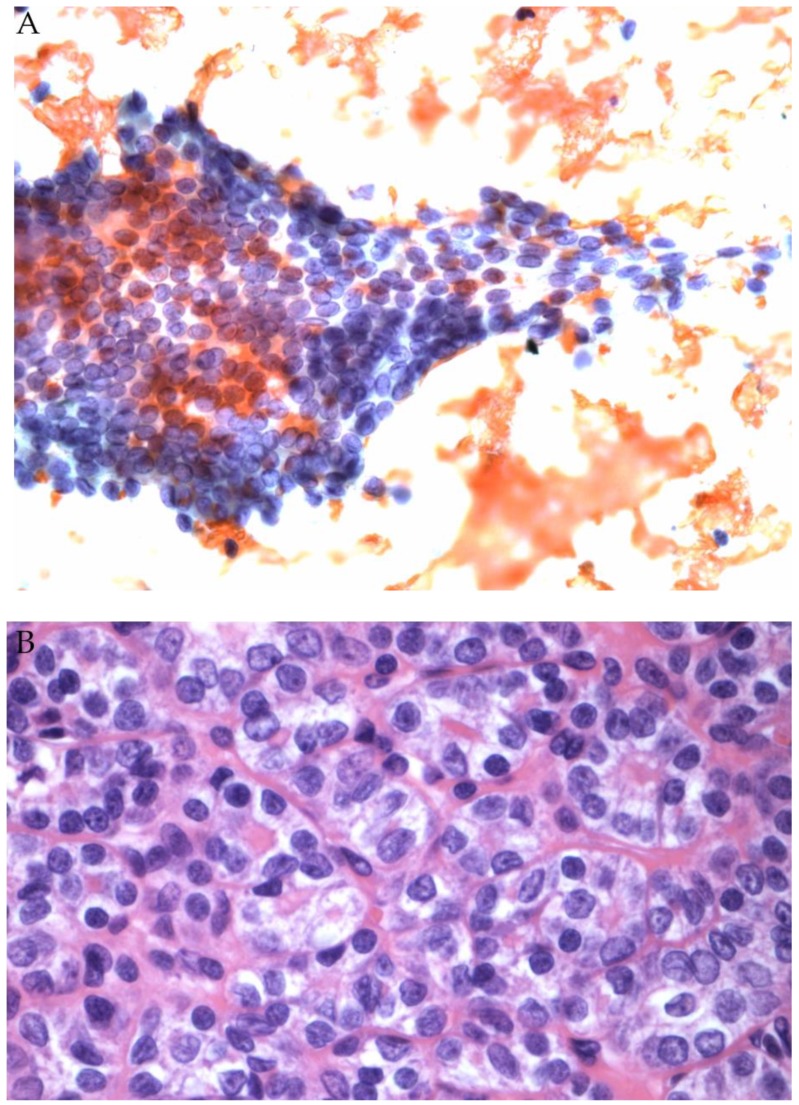
(**A**) FNA of a thyroid nodule classified as AUS/FLUS (Papanicolaou stain). Afirma testing was Suspicious. (**B**) Histology revealed NIFTP (H&E stain).

As stated in TBSRTC, a definitive diagnosis of NIFTP is not possible based upon the cytomorphologic features found in thyroid FNA samples. However, the presence of nuclear pseudoinclusions and papillary cytoarchitecture which are typical features of classical PTC can be used to exclude NIFTP. A predominant follicular pattern and less frequent nuclear elongation and grooves increase the likelihood that the lesion sampled by FNA will be NIFTP [56,98,99,100,101,102,103,104,105,106,107]. Given the prevalence of NIFTP among indeterminate thyroid FNA samples, caution should be exercised to avoid overtreatment. The evaluation of somatic mutations and/or chromosomal rearrangements have shown that NIFTP has a molecular profile that usually differs from classical PTC. In fact, while certain molecular features such as *BRAF^v600E^* mutation are diagnostic of PTC, specific molecular signatures are not available for NIFTP (Figure 3). Despite the absence of any specific genetic alterations linked with NIFTP, molecular testing has been used to distinguish potential cases of NIFTP from other neoplasms [108,109,110,111,112,113]. In general, molecular testing strategies including Afirma GSC and Thyroseq v.3 will likely identify potential FNA cases of NIFTP as atypical, and will triage patients for surgery [108,109,110,111,112,113].

Like encapsulated FVPTC, NIFTP has a different molecular profile from classical PTC [113,114,115]. However, the distinction between invasive forms of FVPTC and NIFTP (i.e., encapsulated non-invasive FVPTC) can only be made based upon histomorphological evaluation. In a study by Kim TH et al., including 177 consecutive FVPTCs (74 non-invasive encapsulated, 51 invasive encapsulated, 52 infiltrative), they showed that any type of *RAS* mutation (*NRAS, HRAS* and *KRAS* mutations) was observed more often in encapsulated FVPTC (48.6% in non-invasive and 66.7% in invasive) and NIFTP than in infiltrative FVPTC (15.4%). Concerning *BRAF^V600E^* mutation, its identification in a thyroid FNA sample can be used to exclude NIFTP. This is also true for *RET-PTC* rearrangements. Molecular features of NIFTP include alterations in *RAS*, *PAX8/PPAη_o_* or *BRAF^K601E^*, in contrast to the frequent *BRAF^V600E^* and *RET/PTC* alterations observed in classical PTC. For this reason, molecular testing such as ThyroSeq v3, the Afirma Xpression Atlas, or ThyGenX could serve as a guide in selected thyroid cases for surgical management (total thyroidectomy vs. hemithyroidectomy).

## 3. Conclusions

The diagnosis of indeterminate lesions of the thyroid is a challenge in cytopathology practice. The evaluation of morphological features alone is unable to provide definitive classification in many cases. The application of ancillary molecular testing for indeterminate thyroid FNA specimens has provided better stratification and triage of patients. Several mutation analysis panels are not only helpful as diagnostic tests, but may also serve as prognostic markers. Much progress has been made in the continual development of molecular testing platforms including the Afirma GSC & Xpression Atlas, ThyroSeq v.3, and ThyGenX/ThyroMIR. Each test has different advantages and limitations in the evaluation of indeterminate thyroid FNA samples. As these molecular tests are improved further making them more accurate and less expensive, they will continue to become a more integral part of the thyroid nodule evaluation.
